# Simple Signaling Molecules for Inductive Bone Regenerative Engineering

**DOI:** 10.1371/journal.pone.0101627

**Published:** 2014-07-14

**Authors:** Emily K. Cushnie, Bret D. Ulery, Stephen J. Nelson, Meng Deng, Swaminathan Sethuraman, Stephen B. Doty, Kevin W. H. Lo, Yusuf M. Khan, Cato T. Laurencin

**Affiliations:** 1 Institute for Regenerative Engineering, University of Connecticut Health Center, Farmington, Connecticut, United States of America; 2 Raymond and Beverly Sackler Center for Biological, Physical and Engineering Sciences, University of Connecticut Health Center, Farmington, Connecticut, United States of America; 3 Department of Chemical Engineering, University of Virginia, Charlottesville, Virginia, United States of America; 4 Department of Orthopaedic Surgery, University of Connecticut Health Center, Farmington, Connecticut, United States of America; 5 School of Medicine, University of Connecticut Health Center, Farmington, Connecticut, United States of America; 6 Center for Nanotechnology & Advanced Biomaterials, School of Chemical & Biotechnology, SASTRA University, Thanjavur, Tamil Nadu, India; 7 Hospital for Special Surgery, New York, New York, United States of America; 8 Department of Medicine, Division of Endocrinology, University of Connecticut Health Center, Farmington, Connecticut, United States of America; 9 Department of Materials Science and Engineering, University of Connecticut, Storrs, Connecticut, United States of America; 10 Department of Chemical and Biomolecular Engineering, University of Connecticut, Storrs, Connecticut, United States of America; University of California, Merced, United States of America

## Abstract

With greater than 500,000 orthopaedic procedures performed in the United States each year requiring a bone graft, the development of novel graft materials is necessary. We report that some porous polymer/ceramic composite scaffolds possess intrinsic osteoinductivity as shown through their capacity to induce *in vivo* host osteoid mineralization and *in vitro* stem cell osteogenesis making them attractive synthetic bone graft substitutes. It was discovered that certain low crystallinity ceramics partially dissociate into simple signaling molecules (i.e., calcium and phosphate ions) that induce stem cells to endogenously produce their own osteoinductive proteins. Review of the literature has uncovered a variety of simple signaling molecules (i.e., gases, ions, and redox reagents) capable of inducing other desirable stem cell differentiation through endogenous growth factor production. Inductive simple signaling molecules, which we have termed inducerons, represent a paradigm shift in the field of regenerative engineering where they can be utilized in place of recombinant protein growth factors.

## Introduction

It is estimated that 50% of Americans will suffer from a traumatic bone fracture that requires orthopaedic services before the age of 65 [1], many of which will require the use of bone grafts such as autografts (the patient's own tissue) or allografts (another person's tissue). Unfortunately, autografts are of limited supply and can cause significant pain at the graft donor site [2], [3], and allografts have the potential to transmit disease and induce an undesirable immune response [4], [5]. To overcome these issues, bone graft substitutes have been heavily researched and are now utilized in approximately 18–20% of all grafting procedures [6]. Some of the most clinically successful bone graft substitutes utilize scaffold-based systems for the spatio-temporally controlled delivery of recombinant human bone morphogenetic proteins (rhBMPs) [7], [8]. While promising, rhBMPs have been plagued by concerns associated with their high cost (in some cases $5,000 a dose) [9], supraphysiological dosage requirements (∼1 million times physiological concentrations) [10], unwanted immunological reactions [11], and undesirable ectopic tissue development [12]. By engineering novel materials-based systems capable of adjuvanting the effects of rhBMPs or replacing them all together, clinical outcomes can be significantly improved while also greatly decreasing costs.

One materials-based approach is to create scaffolds that replicate the organic (i.e. protein) and inorganic (i.e. ceramic) components of native bone. By choosing degradable materials, scaffolds can be fabricated that provide initial support for infiltrating host cells but can be resorbed and replaced as the host regenerates its own tissue. Polymers are suitable organic-phase substitutes since they are biocompatible and possess similar tensile strength to natural bone. Poly(lactide-co-glycolide) (PLGA) is a commonly utilized hydrolytically degradable polymer that is osteocompatible [13] and has been cleared by the Food and Drug Administration for many indications. Hydroxyapatite (HA) is a calcium phosphate (CaP) which is the main inorganic component of bone [14]. It possesses high compressive strength [15] and is osteoconductive (allows for bone cell attachment, migration, and growth) [16] making it an ideal inorganic material for bone tissue engineering. Through the combination of PLGA and HA, we believe that a composite scaffold can be created that capitalizes on the benefits of both materials.

Previously, PLGA and PLGA/HA scaffolds have been fabricated by forming microspheres using emulsion solvent evaporation techniques and melt-sintering them into porous three-dimensional constructs [17]–[19]. PLGA and PLGA/HA scaffolds have been shown to possess mechanical properties similar to trabecular bone making them ideal graft substitutes for bone regenerative engineering applications [13], [17], [19]. Here, *in vivo* and *in vitro* analysis of PLGA/HA scaffolds revealed that these biomaterials possess intrinsic osteoinductivity. The theory we propose for this behavior represents a fundamental change in how regenerative engineering methodologies will be approached henceforth.

## Materials and Methods

### Materials

85∶15 poly(lactide-*co*-glycolide) (PLGA) with a MW of ∼94,000 Da was purchased from Lakeshore Biomaterials (Birmingham, AL). Methylene chloride, calcium nitrate tetrahydrate, ammonium hydrogen phosphate, and polyvinyl alcohol (PVA) (MW ∼70,000 Da) were acquired from Fisher Scientific (Pittsburgh, PA). Ammonium hydroxide, collagenase type I, ascorbic acid, β-glycerophosphate, vitamin D_3_, and cetylpyridinium chloride (CPC) were obtained from Sigma-Aldrich (St. Louis, MO). Dulbecco's Phosphate-Buffered Saline (PBS) and 0.05% trypsin-EDTA solution were purchased from Gibco (Grand Island, NY). Dulbecco's Modified Eagle Medium (DMEM), penicillin-streptomycin solution (Pen-Strep), and fetal bovine serum (FBS) were procured from Invitrogen Corporation (Carlsbad, CA). Triton X-100 was bought from Bio-Rad Laboratories, Inc. (Hercules, CA). Recombinant human bone morphogenetic protein-2 (rhBMP-2) was purchased from GenScript Corporation (Piscataway, NJ).

### Microsphere fabrication

The method for synthesizing PLGA microspheres and PLGA/hydroxyapatite (PLGA/HA) composite microspheres by a double emulsion solvent evaporization technique has been previously described in detail [20]–[23]. PLGA microspheres were fabricated by adding an organic solution of PLGA in methylene chloride (160 mg/mL) via a thin stream into a 1% PVA solution mixed by an overhead stirrer (250 RPM) for 24 hours at room temperature. PLGA/HA microspheres were fabricated by first separately preparing 1.5 mL of 10 M calcium nitrate tetrahydrate (Ca(NO_3_)_2_ • 4 H_2_O) solution and 2.25 mL of 4 M ammonium hydrogen phosphate ((NH_4_)_2_HPO_4_) solution in distilled, deionized water (ddH_2_O). These salt solutions at a calcium/phosphate molar ratio of 1.67 were emulsified together in an organic solution of PLGA in methylene chloride (125 mg/mL) by vortexing. Two different volumes of PLGA solution (24 mL and 12 mL) were used resulting in mass ratios of polymer to ceramic reactants of 40∶60 and 20∶80 which resulted in final polymer/ceramic microsphere compositions of 83/17 PLGA/HA and 73/27 PLGA/HA, respectively. The mixture was added by a thin stream to a 1% PVA solution mixed by an overhead stirrer (250 RPM) for 24 hours at 4°C. A Titrino pH STAT (Metrohm, Herisau, Switzerland) was used to add ammonium hydroxide solution to the emulsion solution in order to maintain a pH of 10. Solidified microspheres were isolated from surfactant solution via decantation and vacuum filtration, rinsed with ddH_2_O, and lyophilized overnight. Dried microspheres were sieved to a diameter range of 355–600 µm using stainless steel sieves (Newark Wire Cloth Company, Clifton, NJ) since microspheres in this size range have been shown to yield scaffolds with a desirable pore size range of 100–350 µm [17]. Microspheres were then stored under desiccant until further use.

### Three-dimensional porous scaffold fabrication

For scaffold fabrication, 355–600 µm microspheres were loaded into a 5 mm×10 mm stainless steel mold and compressed with a piston to achieve desired packing. The mold was heated at 90°C for 90 minutes to melt sinter microspheres together at their intersection points. Scaffolds were allowed to cool to room temperature slowly and then removed from the mold. Scaffolds fabricated for *in vitro* experiments were cut in half (5 mm×5 mm) using a microtome blade. Scaffold morphology was assessed by scanning electron microscopy (SEM) using a JEOL JSM-6335F (JEOL Ltd., Tokyo, Japan) as described previously [17]. Scaffolds were stored under desiccant until further use. Immediately before being utilized for *in vivo* and *in vitro* experiments, scaffolds were sterilized to prevent contamination by being submerged in 70% ethanol, washed with ddH_2_O, and exposed to UV light.

### Ulnar non-union defect and radiographical analysis

Surgical procedures as well as pre- and post-surgical care were performed as specified by Animal Care and Use Committee approved protocols similarly to previously reported methods [18], [24]. Male New Zealand White rabbits (4–5 kg) were anesthetized with a cocktail of ketamine, xylazine, and acepromazine. The right forelimb of the animal was shaved and aseptically prepared with betadine and 70% ethanol. An incision (∼15 mm) was made above the ulna and soft tissue was resected away from the bone. A 10 mm ulnar segment was removed using an oscillating saw irrigated with saline. PLGA, 83/17 PLGA/HA, or 73/27 PLGA/HA scaffolds (5 mm×10 mm, N = 10) were implanted into the defect site. The wound was closed in layers with degradable sutures used for the deep tissue layers and wound clips used for the skin. Pain was managed by pre-operative and post-operative injections of Buprenex and a Duragesic patch (25 ug) for the first 72 hours after surgery. Animals were given prophylactic doses of Baytril (Enrofloxacin) the day before surgery, the day of surgery, and the three days following surgery. Animals were fed ad-libitum and permitted to roam freely within their cages after surgery. The radius acts as a natural splint for the ulna, so no external fixation was used to secure the ulna. Radiographs were taken of the scaffold implanted ulnae immediately after surgery and every four weeks for the duration of the study with an OEC 8800 Digital Imaging System (GE Healthcare, Little Chalfont, UK) to track healing patterns.

### Histological analysis

Animals were euthanized at 8 weeks post-operation and limbs were prepared for histology. Limbs were immersed in 10% formalin for 5 days, soft tissue was resected away from the bone, and the ulna was cut 5 mm proximal and distal to the implanted scaffolds. Samples were then placed in fresh 10% formalin for an additional 48 hours and transferred to 50% ethanol for storage. For sectioning, samples were cold embedded in polymethylmethacrylate (PMMA) for 3–8 days at −20°C to minimize PMMA exothermic curing effects. Embedded sections were cut into 10 µm thick sections and mounted on glass slides. Tissue sections were stained with Von Kossa stain to identify areas of mineralization (black) with a cellular counter stain (neutral red). Histological samples were visualized using an Axiovert 135 light microscope (Carl Zeiss AG, Oberkochen, Germany) and images were taken using a MicroFire digital microscope camera (Optronics, Goleta, CA).

### Adipose derived mesenchymal stem cell (MSC) isolation and culture

Cellular isolation procedures were performed as specified by protocols approved by the University of Virginia Internal Review Board. Human infrapatellar fat pads were obtained from patients undergoing routine total knee arthroplasties at the University of Virginia Hospital. Samples were moved to a sterile cell culture hood where they were rinsed with filter sterilized PBS and minced. An equal volume of 0.1% collagenase type I solution was added to minced sample suspensions and allowed to incubate on a tilting table for 45 minutes at 37°C. Following digestion, the collagenase was neutralized with FBS. The infranatant was transferred to a new tube and centrifuged at 1,200 g for 10 minutes. The fat-enriched supernatant was discarded leaving behind a cell pellet. The pellet was resuspended in DMEM containing 10% FBS and passed through a 100 µm nylon mesh cell filter (Fisher Scientific, Pittsburgh, PA). The filtrate was collected and centrifuged at 12,000 g for 10 minutes. The supernatant was discarded leaving behind a cell pellet rich in adipose derived MSCs. The MSCs were resuspended in growth media (DMEM supplemented with 10% FBS, 100 units/mL Penicillin, and 100 µg/mL Streptomycin) and then plated on T-75 cell culture flasks (Becton Dickinson, Franklin Lakes, NJ) with growth media changed every 72 hours. Cells were grown to ∼70% confluency, lifted from flasks by exposure to 0.05% trypsin-EDTA solution, counted using a hemocytometer, and then passed to new flasks. After 5 passages, MSCs were cryogenically frozen in liquid nitrogen in aliquots of 1 million cells per vial for later use. When a cell study was to be started, frozen MSCs were thawed in a 37°C water bath, diluted in growth media, transferred to T-75 flasks, and cultured as previously described. Cells were passed once and then suspended at a stock concentration of 1.67 million cells per mL for *in vitro* cell studies.

### *In vitro* adipose derived mesenchymal stem cell (MSC) seeded scaffold studies

PLGA, 83/17 PLGA/HA, and 73/27 PLGA/HA scaffolds (5 mm×5 mm) were placed in 24 well plates (Becton Dickinson, Franklin Lakes, NJ), and carefully loaded with 30 uL of stock MSC solution (50,000 cells). MSCs were allowed to adhere to the scaffolds for 10 minutes and then cellularized scaffolds were transferred to new 24 well plates with 1 mL of osteogenic media (DMEM supplemented with 10% FBS, 100 units/mL Penicillin, 100 µg/mL Streptomycin, 50 µM ascorbic acid, 10 µM β-glycerophosphate, and 10 nM vitamin D_3_). Tissue cultured polystyrene (TCP) was utilized as a control and also loaded with 50,000 MSC per well in 1 mL osteogenic media. MSCs were cultured for up to 21 days at 37°C and 5% CO_2_ and media was changed every 72 hours. Cellular morphology at day 21 was analyzed using SEM. Proliferation, protein secretion, and mineralization were assessed at 3, 7, 14, and 21 days. A sample size of 4 was used for all experiments.

### Cellular morphology and proliferation assay

Prior to imaging, cellularized scaffolds were exposed to series dilutions of gluteraldehyde (1% for 1 hour and 3% for 24 hours) and ethanol (30%, 50%, 70%, and 100% for 1 hour each), and then air-dried for 24 hours. Scaffolds were analyzed by SEM in triplicate at different magnifications. Additionally, at each time point, media was removed from the wells and the contents were rinsed with PBS. Scaffolds were transferred to new well plates to ensure that cells not adhered to the scaffolds were not counted. Following the rinse, cells were exposed to 1 mL of 1% Triton X-100 and treated with 3 freeze-thaw cycles to induce complete cell lysis. Lysates were analyzed by the Quant-iT PicoGreen dsDNA Assay (Molecular Probes, Invitrogen, Eugene, OR) using a Synergy HT microplate reader (Biotek, Winooski, VT) at an excitation wavelength of 480 nm and an emission wavelength of 520 nm. Fluorescence was converted to cell number using an adipose derived MSC cell count standard (0–800,000 cells/mL).

### Osteocalcin (OCN) and Bone Morphogenetic Protein-2 (BMP-2) secretion assays

Twenty-four hours prior to each time point, media was removed from the wells, samples were washed with PBS, and fresh media without FBS was added. Scaffolds were transferred to new well plates to ensure that only protein production from scaffold adhered cells would be quantified. At each time point, the FBS-free media was removed from the samples and stored at -20°C until analyzed for protein production. An intact human OCN enzyme-linked immunosorbent assay (ELISA) kit (Biomedical Technologies Incorporated, Stoughton, MA) was used to quantify cell secreted OCN. The assay product solution was visualized using a Biotek Synergy HT microplate reader at a wavelength of 450 nm. The absorbance was converted to OCN concentration with the use of a kit provided human OCN standard (0–50 ng/mL) and then was normalized by cell count. An intact human Quantikine BMP-2 ELISA kit (R&D Systems, Minneapolis, MN) was used to quantify cell secreted BMP-2. The assay product solution was visualized using a Biotek Synergy HT microplate reader at a wavelength of 450 nm. The absorbance was converted to BMP-2 concentration with the use of a kit provided human BMP-2 standard (0–4,000 pg/mL) and then was normalized by cell count.

### Mineralization Assay

At each time point, media was removed from the wells, the contents were rinsed with ddH_2_O, and fixed in 70% ethanol for 24 hours. Scaffolds were transferred to new wells plates to ensure that only scaffolds associated mineralization would be quantified. Acellular scaffolds were exposed to the same culture conditions as the cellularized scaffolds in order to determine the extent of background mineralization due to scaffold-based and/or precipitated calcium phosphate. Following fixation, ethanol was removed and the samples were incubated in 1 mL of 40 mM alizarin red solution (pH 4.23) which binds to calcium phosphate. After 10 minutes, the samples were thoroughly rinsed with ddH_2_O until all non-adsorbed stain was removed. When completely air dried, stained scaffolds were imaged using a Stereo Discovery V12 stereoscope (Carl Zeiss AG, Oberkochen, Germany). Sample associated alizarin red was desorbed using 1 mL of 10% CPC solution (pH 7.0) and concentration was analyzed using a Biotek Synergy HT microplate reader at a wavelength of 550 nm. Absorbance was converted to alizarin red concentration using a standard curve in the linear range (0–0.2740 mg/mL). Sample concentrations that were above the linear absorption range were serially diluted with CPC until they could be quantified. Cell-based mineral deposition was calculated as the difference in mineralization between acellular and cellularized scaffolds and normalized by cell count.

### Scaffold ion release study

PLGA, 83/17 PLGA/HA, and 73/27 PLGA/HA scaffolds (5 mm×5 mm) were loaded in polystyrene microcentrifuge tubes (Thermo Fisher Scientific, Waltham, MA) with 1 mL calcium-free PBS and incubated on a tilting table at 37°C. Aliquots of supernatant (0.5 mL) were collected daily and replaced with fresh PBS for 21 days. Sample calcium ion content was assessed by a Calcium (CPC) Liquicolor Assay Kit (Stanbio Laboratories, Palo Alto, CA) and cumulative calcium ion release was plotted as a function of time.

### Scaffold rhBMP-2 adsorption

Lyophilized rhBMP-2 was reconstituted in 20 mM acetic acid and then used to prepare 30, 125, and 500 µg/mL rhBMP-2 loading solutions in aqueous glutamate-based buffer (5 mM glutamate, 0.5% sucrose, 2.5% glycine, 0.01% Tween 80, pH 4.5). PLGA and 83/17 PLGA/HA scaffolds were sterilized by being submerged in 70% ethanol and then washed with sterile ddH_2_O. Protein solutions (30 µL) were slowly dripped on to corresponding scaffolds yielding final rhBMP-2 loadings of 0.9 µg, 3.75 µg, and 15 µg. Protein loaded scaffolds were allowed to air dry in a sterile cell culture hood, lyophilized for 24 hours, and sterilized by exposure to UV light.

### *In vitro* MSC seeded rhBMP-2 loaded scaffolds studies

PLGA scaffolds, rhBMP-2 (0.9 µg, 3.75 µg, or 15 µg) loaded PLGA scaffolds, 83/17 PLGA/HA scaffolds, rhBMP-2 (0.9 µg, 3.75 µg, or 15 µg) loaded 83/17 PLGA/HA scaffolds, and TCP were seeded with MSCs as described for the previous study and cultured in osteogenic media for up to 21 days. Proliferation, OCN secretion, and mineralization were assessed at 3, 7, 14, and 21 days as described above. Cell secreted BMP-2 was determined as the difference between the BMP-2 measured for acellular and cellularized scaffolds using a BMP-2 ELISA kit.

### Cumulative BMP-2 release study

PLGA and 83/17 PLGA/HA scaffolds were either seeded with MSCs or loaded with rhBMP-2 as described above and cultured in osteogenic media for 21 days. Each day, media was removed from the samples, stored at −20°C, and replaced with fresh media. After 21 days, all samples were analyzed for their BMP-2 content using a BMP-2 ELISA kit. The kit provided human BMP-2 standard (0–4,000 pg/mL) was used to quantify cell secreted BMP-2, whereas a rhBMP-2 standard (0–4,000 ng/mL) was used to quantify protein released from the rhBMP-2 loaded scaffolds. Cumulative BMP-2 was plotted as a function of time.

### Statistical analysis

All results in the figures are reported as mean ± standard deviation. JMP software (SAS Institute, Cary, NC) was used to make comparisons between groups using an ANOVA followed by Tukey's HSD test to determine pairwise statistically significant differences (p<0.05).

## Results

### Composite scaffold-mediated *in vivo* neo-bone formation

**Figure 1:**
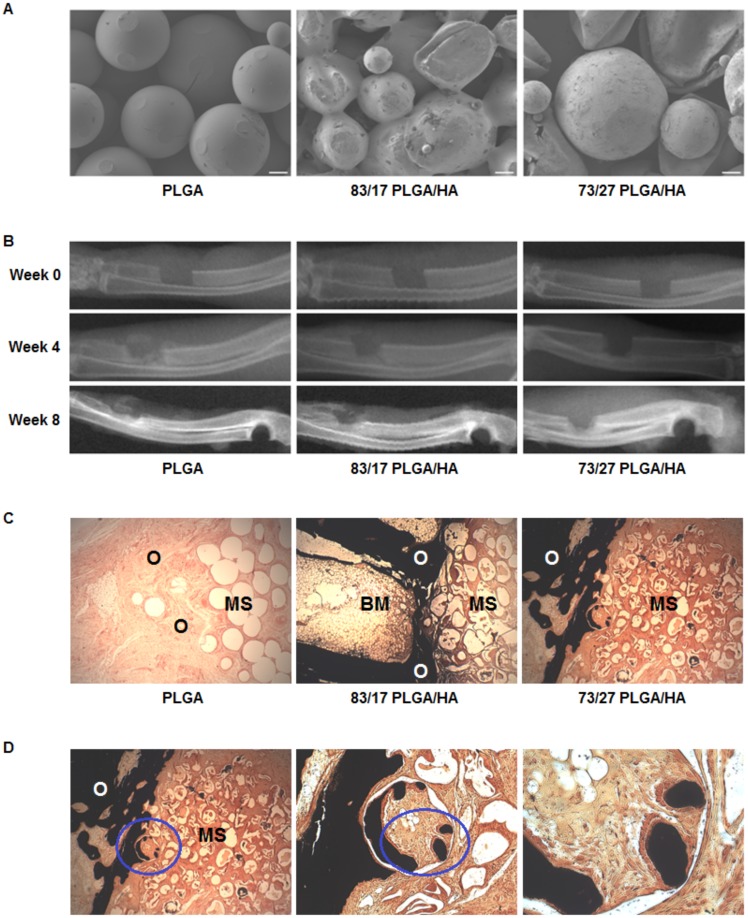
*In vivo* osteoinductivity of PLGA/HA composite scaffolds. (**A**) Representative scanning electron micrographs of sintered microsphere scaffolds (Scale Bar  = 100 µm). (**B**) Representative radiographs of 10 mm rabbit ulnar non-union segmental defects loaded with different scaffold compositions. (**C**) Optical micrographs of histological sections stained with von Kossa stain at 8 weeks (MS =  Microsphere Scaffold, O =  Osteoid, BM =  Bone Marrow; Magnification  = 40x). (**D**) Von Kossa staining of the histological sections for the 73/27 PLGA/HA composite scaffolds show the incorporation of microspheres into newly forming mineralized osteoid bone. The blue ellipsoid signifies the next field of view; Magnification  = 40x (left), 100x (center), and 320x (right).

PLGA/HA composite scaffolds were created by first precipitating HA into PLGA microspheres *in situ* using a double emulsion solvent evaporation technique and then melt-sintering the microspheres into three-dimensional cylindrical scaffolds as described previously [19]. Scaffolds composed of PLGA and two different compositions of PLGA/HA (83/17 and 73/27 weight ratios) were evaluated in this research. Scanning electron microscopy revealed porous three-dimensional scaffolds comprised of microspheres with melted intersection points regardless of scaffold composition (Fig. 1A). Microtopographically, PLGA scaffolds had a smooth surface whereas both PLGA/HA scaffolds possessed a rough surface attributable to the *in situ* precipitated HA present on the microsphere surface. All three compositions of scaffolds were implanted in rabbit ulnar segmental non-union defects and radiographs were taken throughout the healing process (Fig. 1B). Immediately after implantation (0 weeks), the defect was clearly visible in the mid-region of the ulna. At 4 weeks post-implantation, the defects showed signs of early healing at the scaffold-bone interface. By 8 weeks post-implantation, radiographic evidence of mineralized callus formation was seen as well as complete bridging along the medial half of the defect for both the PLGA and 83/17 PLGA/HA scaffolds. For the 73/27 PLGA/HA scaffolds, abundant healing along the medial half of the defect with nearly complete bridging was seen. These results provide evidence that bone healing proceeded across the defect regardless of scaffold type. Animals were euthanized at 8 weeks and tissue samples were sectioned and stained with von Kossa stain. Histological analysis of resected samples showed tissue ingrowth throughout the microsphere structure of all scaffolds regardless of composition (Fig. 1C). However, there were significant differences between the mineralization seen in PLGA scaffolds compared to both PLGA/HA scaffolds. PLGA scaffolds showed no dark staining at the host interface indicating a lack of mineralized tissue. In contrast, both PLGA/HA scaffolds showed significant positive mineral staining at their host interfaces as well as within the edges of the scaffold interior. Specifically, the 73/27 PLGA/HA scaffolds showed incorporation of the microsphere matrix by the host osteoid tissue (Fig. 1D). Mononuclear cells were found in a band along von Kossa stained calcium phosphate (CaP) analogous to osteoblasts lining up along mineralizing fronts of remodeled bone tissue. This behavior was not seen with the PLGA scaffolds and is similar to results seen with other polymer/CaP constructs [25], [26] suggesting that the HA component in PLGA/HA scaffolds can induce new mineralized bone growth without the inclusion of exogenous growth factors or cells.

### Composite scaffold-mediated *in vitro* stem cell differentiation

**Figure 2:**
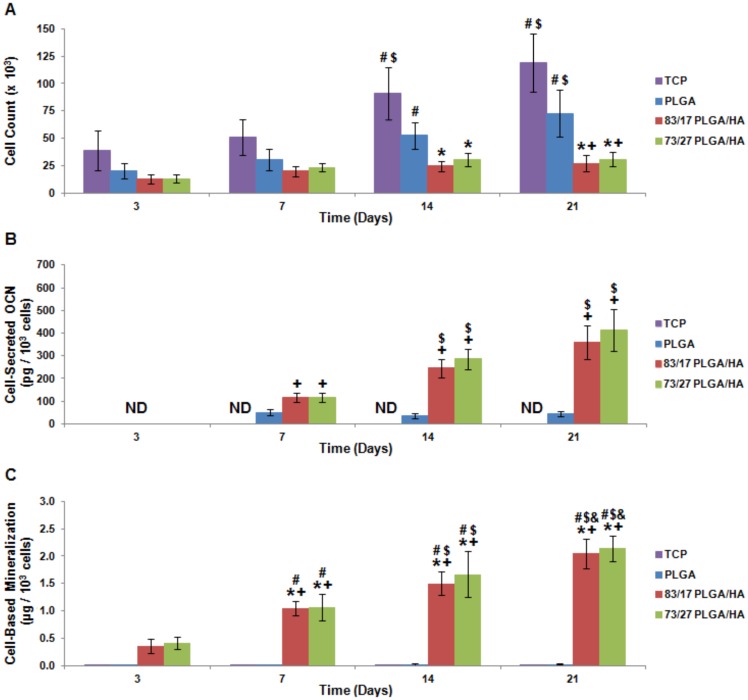
*In vitro* osteoinductivity of PLGA/HA composite scaffolds. (**A**) MSC proliferation as measured by the Quant-iT PicoGreen Assay. (**B**) Mineralization as assessed using alizarin red staining of deposited calcium. Cell-based mineralization was quantified by subtracting scaffold-based mineralization and normalizing by cell count. (**C**) MSC OCN production induced by seeding on PLGA/HA scaffolds. Cell-secreted OCN as determined by ELISA and normalized by cell count. ND =  Not Detectable; N = 4; p<0.05 for + =  PLGA (same time point) and $ =  Same Experimental Group (Day 7).

The osteoinductivity of PLGA/HA scaffolds was studied further through *in vitro* experimentation using human adipose-derived mesenchymal stem cells (MSCs). Human adipose-derived MSCs were chosen since they are easy to extract from the host and culture into highly pure populations [27]. MSCs were seeded and cultured for up to 21 days on tissue culture polystyrene (TCP), PLGA scaffolds, 83/17 PLGA/HA scaffolds, or 73/27 PLGA/HA scaffolds. Scanning electron microscopy confirmed that MSCs were able to span scaffold pore spaces by utilizing filopodia to attach to microsphere surfaces and were found to be evenly distributed throughout the scaffolds regardless of scaffold composition (data not shown). At 3, 7, 14, and 21 days, cells were assessed for their proliferation and osteogenic differentiation. MSCs cultured on TCP or PLGA scaffolds showed statistically significant increases in cell number over time, whereas cells cultured on either of the PLGA/HA scaffold compositions showed limited increases in cell number over time (Fig. 2A). By 21 days, significantly higher cell counts were found for MSCs seeded on TCP or PLGA scaffolds than those seeded on PLGA/HA scaffolds. Lower cellular proliferation on PLGA/HA scaffolds suggests potential cellular differentiation. In order to determine if MSCs were differentiating down the osteoblastic lineage, cellular secretion of the pro-osteoblastic protein osteocalcin (OCN) and cell-based mineral deposition were analyzed. OCN secretion was assayed by an enzyme-linked immunosorbent assay (ELISA) and significant differences were seen between the experimental groups (Fig. 2B). MSCs grown on TCP and PLGA scaffolds had non-detectable and barely detectable levels of OCN secretion, respectively. In contrast, MSCs grown on either of the PLGA/HA scaffold compositions showed statistically significant increases in OCN secretion over time, providing evidence that the MSCs were differentiating into osteoblasts. Mineral deposition was assayed by alizarin red stain, which tightly binds calcium and calcium composites. Sterioscopic images showed alizarin red staining of the CaP on the surface of acellular PLGA/HA scaffolds as expected, but a deeper red color was present on the MSC-seeded PLGA/HA scaffolds, which indicated additional, cell-based, mineral deposition (data not shown). Alizarin red stain was desorbed from the scaffolds and spectrophotometrically analyzed with acellular staining subtracted in order to determine cell-based mineralization. MSCs incubated on either PLGA/HA scaffold composition deposited statistically significant greater quantities of mineral compared to nearly non-detectable levels of mineral deposition by cells incubated on TCP or PLGA scaffolds (Fig. 2C). Together, these *in vivo* and *in vitro* data provide significant evidence that HA containing scaffolds may be intrinsically osteoinductive.

### Intrinsic osteoinductivity of composite scaffolds

**Figure 3:**
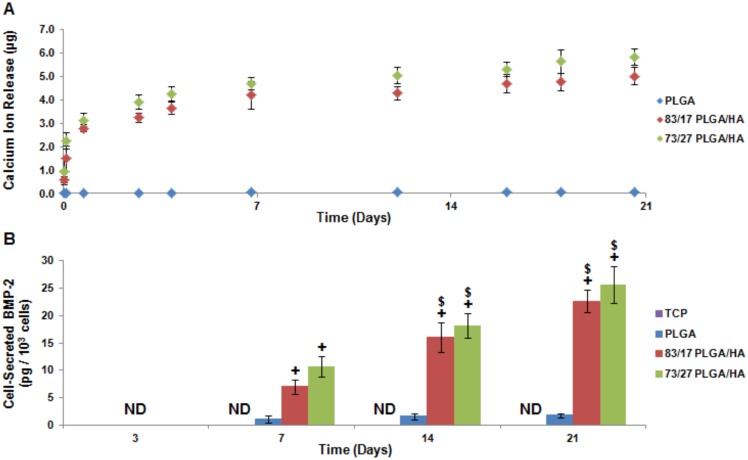
PLGA/HA composite scaffold ion release mediates cell-based BMP-2 production. (**A**) Scaffold-based ion release as determined by the Calcium (CPC) Liquicolor assay. (**B**) BMP-2 secreted from cells as analyzed by ELISA and normalized by cell count. ND =  Not Detectable; N = 4; p<0.05 for * = TCP (same time point), + =  PLGA (same time point), # =  Same Experimental Group (Day 3), $ =  Same Experimental Group (Day 7), and & =  Same Experimental Group (Day 14).

The biological basis of CaP osteoinductivity has yet to be completely characterized. In fact, some CaPs, like highly crystalline HA, have been found to possess limited intrinsic osteoinductivity [28]. The prevailing CaP osteoinduction theories focus around three complementary but disparate ideas: 1) local presence of calcium and phosphate ions, 2) mechanobiological triggering due to surface topography, and 3) scaffold absorption and presentation of endogenously released osteogenic growth factors [29]. Recent research has shown that human periosteal stem cells undergo osteogenic differentiation when exposed to calcium and phosphate ions without any supplemental factors providing strong evidence that the ions themselves possess intrinsic osteoinductivity [30]. The results herein align with other research that has shown CaP ion release correlated to the quality and quantity of new *in vivo* bone formation [28]. *In situ* precipitated HA present in the PLGA/HA scaffolds has been found to be of low crystallinity [19]. When submerged in phosphate buffered saline (PBS), both PLGA/HA scaffold compositions release approximately the same significant quantities of calcium ions whereas PLGA scaffolds showed no ion release (Fig. 3A). We theorized that calcium and phosphate ions released from PLGA/HA scaffolds induce seeded MSCs to produce their own desirable biomolecules. Interestingly, a recent study demonstrated that calcium ions induce bone marrow derived stromal cells to undergo osteogenesis through the ERK1/2 signaling pathway resulting in downstream BMP-2 gene expression [31]. Utilizing a BMP-2 ELISA, it was discovered that MSCs seeded on PLGA/HA scaffolds produced and secreted significant quantities of BMP-2 that increased over time (Fig. 3B). MSCs seeded on PLGA scaffolds were found to secrete low levels of detectable BMP-2 which is most likely due to the small quantities of calcium and phosphate ions, β-glycerophosphate, ascorbic acid, and Vitamin-D_3_ found in the media. To our knowledge, CaP induction of cell-based bone morphogenetic protein production has not been previously shown in the literature. This data provides strong evidence that calcium and phosphate ions act as simple signaling molecular triggers capable of inducing a cell-based BMP-2 autocrine and paracrine osteoinduction loop.

### Growth factor addition does not enhance scaffold osteoinductivity

**Figure 4:**
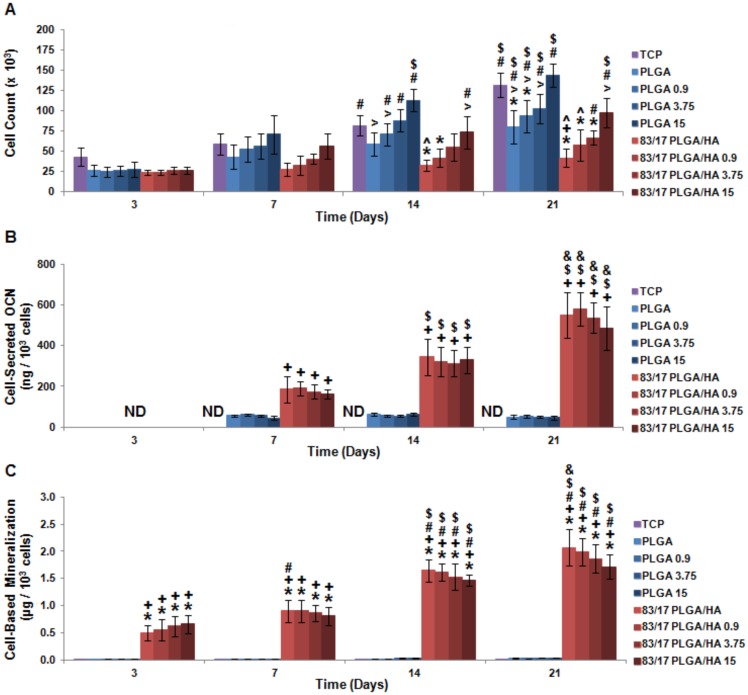
*In vitro* osteoinductivity of BMP-2 loaded PLGA/HA composite scaffolds. (**A**) MSC proliferation as measured by Quant-iT PicoGreen Assay. (**B**) Mineralization as assessed by alizarin red staining of deposited calcium. Cell-based mineralization was quantified by subtracting out scaffold-based mineralization and normalizing by cell count. (**C**) MSC OCN production induced by BMP-2 loaded PLGA/HA scaffolds. Cell-secreted OCN determined by ELISA and normalized by cell count. ND =  Not Detectable; N = 4; p<0.05 for + =  PLGA (same BMP-2 loading and same time point), $ =  Same Experimental Group (Day 7), and & =  Same Experimental Group (Day 14).

To further investigate materials-based osteoinductivity, rhBMP-2 was surface-adsorbed to the scaffolds. By loading rhBMP-2 onto both PLGA and PLGA/HA scaffolds, the comparative and synergistic osteoinductive effects of HA and rhBMP-2 were studied. Since ion release and osteoinductivity for both the PLGA/HA scaffolds compositions were found to be highly similar, only the more mechanically competent 83/17 PLGA/HA [19] was utilized for this study. Different dosages (0.9 µg, 3.75 µg, or 15 µg) of rhBMP-2 were first surface-adsorbed onto either PLGA or 83/17 PLGA/HA scaffolds by lyophilization. MSCs were then seeded onto TCP, PLGA scaffolds, rhBMP-2 loaded PLGA scaffolds, 83/17 PLGA/HA scaffolds, or rhBMP-2 loaded 83/17 PLGA/HA scaffolds and assessed for their cell proliferation, protein production, and mineralization at 3, 7, 14, and 21 days. MSCs seeded on either scaffold composition had higher cell counts correlating to increased rhBMP-2 loading which became statistically significant over time (Fig. 4A) and is consistent with the role of rhBMP-2 as a mitogen [32]. MSC osteogenesis was analyzed by OCN secretion and scaffold mineralization. Overall cell-secreted OCN production increased slightly according to a trend of increasing rhBMP-2 quantity, but these differences disappeared when protein production was normalized by cell count (Fig. 4B). For cell-based mineralization, a similar trend was seen where the slight effect of rhBMP-2 loading was negated by cell count normalization (Fig. 4C). For both OCN secretion and cell-based mineralization, MSC osteogenesis was directly related to the presence of HA in the scaffolds and could not be induced or enhanced by the incorporation of rhBMP-2.

**Figure 5:**
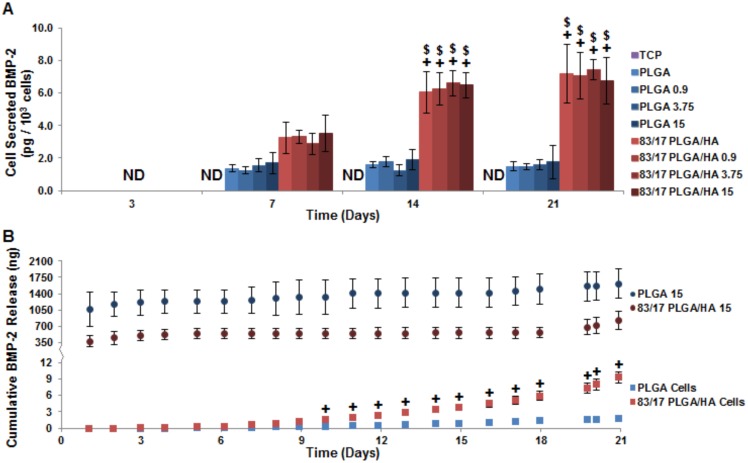
Cell-based BMP-2 production unaltered by loading exogenous BMP-2 on scaffolds. (**A**) Total BMP-2 as analyzed by ELISA and normalized by cell count. (**B**) Cumulatively released BMP-2 from protein-loaded scaffolds (top) or MSCs seeded on protein-free scaffolds (bottom). ND =  Not Detectable; N = 4; p<0.05 for * =  TCP (same time point), + =  PLGA (same BMP-2 loading and same time point), > =  PLGA 15 (same time point), ∧ = 83/17 15 (same time point), # =  Same Experimental Group (Day 3), $ =  Same Experimental Group (Day 7), and & =  Same Experimental Group (Day 14).

The effect of scaffold loaded rhBMP-2 on MSCs to produce their own BMP-2 was also investigated in this study. Total BMP-2 was quantified using a BMP-2 ELISA and cell-based BMP-2 production was determined by subtracting out the rhBMP-2 release from acellular scaffolds (Fig. 5A). The presence of large supraphysiological quantities of rhBMP-2 on the scaffolds was unable to induce MSCs to increase endogenous production of BMP-2. Protein release from rhBMP-2 loaded acellular scaffolds (PLGA 15 and 83/17 PLGA/HA 15) or secreted from rhBMP-2 free cellular scaffolds (PLGA Cells and 83/17 Cells) were analyzed by BMP-2 ELISA (Fig 5B). Intact rhBMP-2 was released by both scaffold compositions in a triphasic profile of burst (day 1), lag (days 2–17), and secondary release (days 18–21) with PLGA 15 scaffolds releasing two-fold to three-fold greater amounts of rhBMP-2 than 83/17 PLGA/HA 15 scaffolds. This difference in rhBMP-2 release is attributed to the stronger protein binding that occurs to the HA in composite scaffolds than PLGA [23]. Cell-based BMP-2 was not secreted until 3-4 days post-incubation. MSCs seeded on PLGA scaffolds produced low quantities of BMP-2 whereas MSCs seeded on 83/17 PLGA/HA scaffolds showed geometrically increasing BMP-2 secretion over time. By day 10, BMP-2 production from cells seeded on 83/17 PLGA/HA was found to be significantly greater than cells seeded on PLGA. While the burst release of rhBMP-2 may not be ideal for osteoinduction, BMP-2 release during the lag phase and secondary phase was found to be one to three orders of magnitude greater than the largest quantity of cell-based BMP-2 secreted. Additionally, it should be noted that greater than 90% of loaded rhBMP-2 was still adsorbed to both scaffold compositions at Day 21. Since BMPs are capable of carrying out their osteoinductive function without being internalized [33], the rhBMP-2 loaded scaffolds exposed seeded MSCs to at least four orders of magnitude greater quantities of BMP-2 than cells seeded on protein-free 83/17 PLGA/HA scaffolds secreted throughout the experiment. The addition of supraphysiological rhBMP-2 dosages was unable to modulate cell-based BMP-2 production; instead the presence of HA in the PLGA/HA scaffolds was responsible for cell-based BMP-2 secretion.

## Discussion

**Figure 6:**
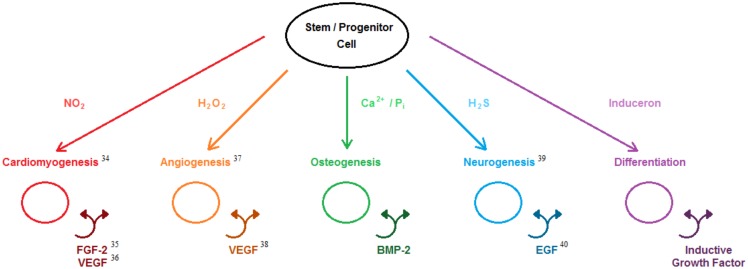
Induceron theory. Inducerons (simple signaling molecules) are capable of inducing stem and progenitor cells to undergo desired differentiation and to produce their own endogenous growth factors. While evidence has been provided in this paper for osteoinductive inducerons, other research has shown that a variety of different inducerons are capable of carrying out other desirable inductive cellular functions.

The results presented in this report corroborate our novel theory that the simple signaling molecules of calcium and phosphate ions possess intrinsic osteoinductivity which is carried out by the induction of cell-based osteoinductive protein growth factor (BMP-2) production and secretion. Review of published biochemical research has revealed that there exist a number of simple signaling molecules that possess similar behavior to calcium and phosphate ions for which we have collectively termed inducerons (Fig. 6). These small molecules are uniquely capable of inducing stem and progenitor cell differentiation down desired lineages utilizing protein growth factor-based inductive autocrine and paracrine loops. The problems associated with using BMP-2 in bone graft substitutes such as cost and long-term safety are also issues with utilizing protein growth factors in other translational biomedical engineering strategies. Since these inductive molecules can be released from stable, inexpensive materials (e.g. CaP), their long-term delivery can be achieved through a wide variety of controlled release strategies compared to the relatively few options available for fragile, expensive protein growth factors. In the United States, more than 115,000 people are currently on the organ transplantation list and over 7,000 people will die each year waiting for viable donor organs to become available [34]. Furthermore, the quality of life for those individuals that do receive transplanted organs is severely hampered by high organ rejection rates (23.7%–57.3% at 5 years post-transplantation) [34] and the side effects associated with immunosuppressive drugs [35]. Utilizing the principles of biology, engineering, morphogenesis, stem cell technology, and materials cues, regenerative engineering represents a novel approach in which the body is induced to regenerate its own complex tissues and organs. This new concept of inducerons may compel regenerative engineering strategies to become the new gold standard in complex tissue and organ replacement therapies.
